# Cognitive Heterogeneous Wireless Network and Artificial Intelligence-Based Supply Chain Efficiency Optimization Application

**DOI:** 10.1155/2022/8482365

**Published:** 2022-07-08

**Authors:** Yuan Yuan

**Affiliations:** ^1^Hebei University of Technology, Tianjin 300401, China; ^2^Hebei GEO University, Shijiazhuang, Hebei 050031, China

## Abstract

Cognitive radio can specifically perceive the surrounding environment, can understand and learn the changes in the surrounding environment, and can adjust its own adaptation method, channel calculation, and other transmission parameters at any time. This cognitive radio method is more flexible and is an intelligent wireless communication system, a relatively advanced system. Cognitive communication devices can detect changes in the surrounding wireless environment and relatively change their own parameters to cope with the changes in the environment and ensure the stability of communication quality. As a different part of the supply chain, enterprises use the sharing economy and cooperation to improve the overall competitiveness of the enterprises in the supply chain, ultimately achieve a win-win effect for all enterprises in the supply chain, and complete the improvement of the company's competitiveness. The supply chain model can help companies to be familiar with the structure, operation process, and orientation of the supply chain in which they are located, so that they can clearly understand the work process through simulation scenarios and better understand the problems of the supply chain work process. Supply chain supply with cognitive heterogeneous wireless networks and artificial intelligence is a necessary way to deeply improve the operation and working process of supply chain control. In short, the cognitive radio system proposed in this study can receive network-related messages by observing the surrounding wireless network environment, and can receive internal communication tasks and emergency communication tasks based on historical data and prediction of future relationships. Through the access control process, we make an appropriate strategy and finally complete the communication function of the system.

## 1. Introduction

Cognitive radio is one of the extended forms of software radio, and the radio uses software radio as the system platform, which is intelligent, very reliable, and adaptable, and is more intelligent than software radio [1]. Cognitive radio can specifically perceive the surrounding environment, has the ability to understand and learn changes in the surrounding environment, and can adjust its own adaptation method, channel calculation, and other transmission parameters at any time [2]. This cognitive radio method is more flexible, intelligent, and very reliable. It has strong adaptability and is a relatively advanced system of the intelligent wireless communication system. Cognitive communication devices can detect changes in the surrounding wireless environment and relatively change their own parameters to cope with the changes in the environment and ensure the stability of communication quality [3].

At present, load dialing algorithms used in heterogeneous wireless networks are specifically divided into two categories: load and balance algorithms based on access control and load-balancing algorithms based on forced transmission [4]. New calls can be connected to and connected to the network. In order to avoid an unbalanced situation after a new call is connected to the system, it may lead to unnecessary waste and an increase in payment [5]. If the threshold is set too high, it is not easy to trigger a loading mechanism when the system is overloaded. The dynamic adjustable threshold is undefined, but it can be dynamically adjusted, and the ice load status of each network in the system will change.

With the rapid evolution of industry and information technology, traditional supply chain management methods have fallen far behind the high requirements set by modern market operation methods [6]. The competition between enterprises has changed the cooperative relationship between supply chains and between groups. Competitive relationship. As a different part of the supply chain, enterprises use the sharing economy and mutual cooperation to improve the overall competitiveness of the enterprises in the supply chain, ultimately achieve a win-win effect for all enterprises in the supply chain, and complete the improvement of the company's competitiveness [7]. The supply chain model can help companies to be familiar with the structure, operation process, and orientation of the supply chain in which they are located, so that they can clearly understand the work process through simulation scenarios and better understand the problems of the supply chain work process [8]. Its improvement will ultimately enhance the supply and the effect of the chain. Supply chain supply with cognitive heterogeneous wireless networks and artificial intelligence is a necessary way to deeply improve the operation and working process of supply chain control [9].

## 2. Related Work

In the literature, it is suggested that supply chain management should cover all parts of the product value chain, and supply chain members should design and plan the benefits of supply chain members through communication and communication to achieve the company's final goal [10]. The viewpoint of supply chain distribution channels refers to the product and service system composed of the front, middle, and back companies [11]. The ultimate goal of the entire supply chain is to meet the requirements of end users. The literature suggests that supply chain management will be an uninterrupted process that should focus on this process rather than specific tasks, and supply chain management should reduce the time and product costs for product entry, ultimately meet customer requirements, and enhance customer satisfaction rate [12]. The literature pointed out that the key ideas of supply chain management are “system thinking” and “river thinking.” The supply chain is an overall system that can efficiently manage the product flow, capital flow, and property flow in the system [13]. Supply chain management has been fully applied in the actual operation of companies in the 21st century. The company has fully integrated information and supply chain management, which has promoted tremendous improvements in supply chain management. Through the core company to create a supply chain model, more and more attention is paid to supply chain management and the unity and collaboration between supply chain companies [14]. In the literature, a supply chain model is proposed, which can help companies analyze the entire supply chain, lay a solid foundation for business decision-making, and ultimately help decision-makers formulate better strategies, which can then efficiently support supply chain management [15]. The research on supply chain models in various countries in the world is very comprehensive; the supply chain management model has been continuously improved and improved; new developments have been made in the research results; and good results have been achieved in practical applications. A summary method of the existing load-balancing algorithms in heterogeneous wireless networks is proposed [16]. An access control algorithm that regards users as the center is proposed. This algorithm uses dynamic pricing to complete load balancing; based on user preferences, the algorithm regards to price as the main aspect of user selection of the network [17]. We use multipurpose decision-making methods to access and select the network that is most convenient for users, by adjusting the price of each wireless network at any time to complete the load balance of the system, and then to meet the user's choice.

## 3. Artificial Intelligence Supply Chain Efficiency Optimization Algorithm Model Design

### 3.1. Cognitive Wireless Network

**Figure 1:**
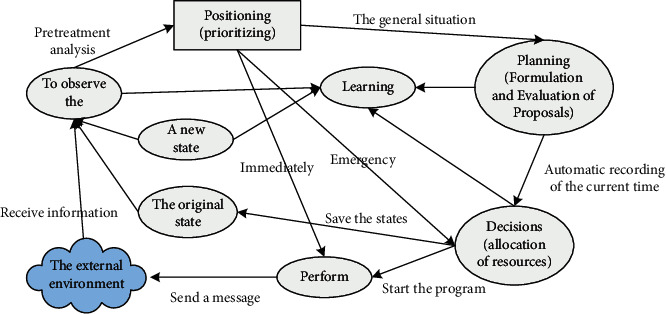
Cognitive cycle model diagram.

Cognitive radio can automatically extract data that are conducive to improving system functions based on past historical results and environmental changes. The cognitive cycle model mentioned by Joseph Mitola can well reflect the concept of cognitive radio. The cycle model mainly includes six parts: environmental observation, self-positioning, planning, intelligent training, deployment, and implementation. The main function of the observation phase is to implement the collection and storage of surrounding wireless environment data. The main function of self-positioning is to determine the level of excellence related to the urgency of communication services, sort users according to the main functions of the planning stage, prepare decision-making plans, evaluate the feasibility of the plan, and plan the learning stage based on relevant information. It is the continuous learning and decision management of the scenarios around the wireless network. The deployment phase is the calculation and distribution of wireless spectrum resources. The most important function of the implementation phase is that the cognitive radio starts to allocate its work according to the resources. The cognitive cycle model diagram is shown in Figure 1.

### 3.2. Heterogeneous Network Model

In the heterogeneous wireless convergence system, different wireless networks use different frequency bands and communication methods, making the channel leasing of the heterogeneous network no longer applicable, and the load balancing of the heterogeneous network must be solved by transferring the load. In terms of access speed and resource types, the burden sharing of heterogeneous wireless systems requires unified management of wireless resources, and load and planning in a large-scale manner. The load-balancing methods in heterogeneous wireless networks include centralized, distributed, and distributed methods. Three types of layers are as follows:

1. The central node and local nodes manage the load information of the network. The task of the central node is to collect the load information data sent by each local node, make a load-balancing decision based on the data, and send it to the local node instruction. The central distribution mechanism can quickly make the best decision based on the overall status, with higher efficiency and less signaling workload. The disadvantage of the centralized load-balancing mechanism is that it is vulnerable to attacks. If the central node fails due to a failure, the entire load-balancing mechanism will not continue.
2. The distribution of load balancing is a distributed load system. Each node has an information table that records the load status of itself and adjacent nodes, and calculates and operates load-balancing decisions based on the load data in the table; if a node fails, the table will not affect the load of other nodes too much. The disadvantage is that each node must exchange a lot of information with the surrounding nodes; the amount of signaling is large, and the time is prolonged.
3. Hierarchical load-balancing control mode hierarchical load-balancing control mode is a combination of collective and distributed modes; combining the advantages of both, this mode has higher security and lower signal input.

When users choose to access the network, they usually choose network access related to network bandwidth, and receive signal information, network cost, etc. from the perspective of personal preference. These are excellent, but it is easy to cause imbalances between networks. There is a phenomenon of the load in the system. Large transportation hubs, such as airports and stations, where Wi-Fi networks are congested due to high traffic, and mobile phone networks have free resources and low loads in their overlapping supply areas, so the utilization rate of network resources in the system is very low. We manage and adjust each network in a heterogeneous system to balance network resources in different networks.

**Figure 2:**
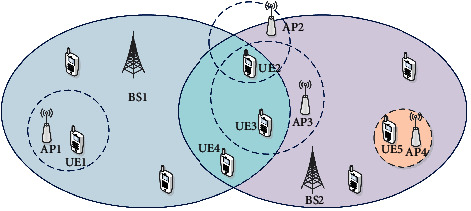
Heterogeneous convergence system of UMTS and WLAN.

The distribution algorithm based on utility function and fuzzy logic pointed out in this study is the use of forced switching load commands. The main idea of this algorithm is to first evaluate the balance of network load. Different from the forced distribution algorithm based on forced handover, this algorithm defines various immediate and nonimmediate use functions of the service, in order to propose a better service level to users and better realize the full utilization of the network. The research model of this algorithm uses a heterogeneous overlay network system based on UMTS and WLAN. The heterogeneous fusion system of UMTS and WLAN is shown in Figure 2:

The load of the network represents the resource allocation rate in the network. This is the most important factor to be considered in the load-balancing algorithm of a heterogeneous network. The main factors are as follows:

(1) $$\begin{array}{c} p=\frac{1}{R}\sum_{i=1}^{M}\frac{GB\left(i\right)SF\left(i\right)R\left(i\right)}{R\left(i\right)}. \end{array}$$

From the user and network point of view, we fully consider the service data, the benefits of user QoS, the use of wireless network resources, and the fair user selection strategy, and the user who will use the device terminal most appropriately will be selected. For the user's QoS, you can select the target user, that is, the user with little satisfaction. We select users with less QoS income as the transfer target. The algorithm uses the sigmoid function to construct the winning function of the terminal. 1 represents the number of all users and specifies the number of all RAN networks. The QoS function is as follows:

(2) $$\begin{array}{c} U_{i,j}=\left\{\begin{array}{cc} \left(1-\frac{1}{1+\exp\left(-\beta \times d_{i,j}-d_{i,j}/d_{i,j}-d_{i,j}\right)}\right), & RT,\\ \frac{1}{1+\exp\left(-\beta \times r_{i,j}-r_{i,j}/r_{i,j}-r_{i,j}\right)}, & \text{NRT}. \end{array}\right. \end{array}$$

The utilization rate of wireless terminals in the current connected network should be calculated. When selecting users, the user with a lower utilization rate of network resources should be selected for handover, and the low-end network activity will be terminated. The resource allocation rate of the terminal in the access network *j* is as follows:

(3) $$\begin{array}{c} P_{i,j}=\frac{R_{i,j}\left(\alpha_{i,j}\right)}{R_{i,j}^{\max}}. \end{array}$$

According to the conditions, we select users with a low total QoS usage rate of the terminal and a low wireless resource usage rate for handover. Therefore, *w* is defined as the user's QoS benefit and the overall benefit of wireless resource usage in the network. Its value is simple to use that is calculated by the weighing method.

(4) $$\begin{array}{c} w_{i,j}=\alpha U_{i,j}+\beta \rho_{i,j}. \end{array}$$

If the user service is a real-time service, we use the service program function together with the real-time service blocking speed and network balance as an input variable in the fuzzy logic system. This is good for the company's real-time blocking rate and can be expressed as follows:

(5) $$\begin{array}{c} U_{RT}\left(j\right)=\frac{1gP_{RT}\left(j\right)}{1gP_{RT\_\max}}. \end{array}$$

Among them, *P*(*j*) is the actual blocking speed of the instant service in network *j*, and *P* represents the maximum blocking speed that the instant service can withstand. The smaller the network overload, the larger the value. The specific representation is as follows:

(6) $$\begin{array}{c} U_{B\left(j\right)}=\frac{\left[\sum_{i=1,i=j}^{K}\left(x_{i}/W_{T,1}\right)+\left(x_{i}+W_{j,m}/W_{T,j}\right)\right]}{K\left[\sum_{i=1,i=j}^{K}{\left(x_{i}/W_{T,i}\right)}^{2}+\left(x_{j}+W_{j,m}/W_{T,j}\right)\right]}. \end{array}$$

In the fuzzy logic system, the use function *U* and the compensation function *B* of the sluice percentage are used as input variables, and the fuzzy logic system is processed to realize the function of the input variables: first, the input variables *U* and *B* must be normalized. For the use of function U, the better the normalization formula, the smaller the use function.

(7) $$\begin{array}{c} {\bar{U}}_{RT}=\frac{U_{MAX}-U_{j}}{U_{MAX}-U_{MIN}}. \end{array}$$

After fuzzing the input variables, the fuzzy output value must be obtained according to the fuzzy inference criterion. According to the barrel principle, the fuzzy emission takes the minimum level of the input variable as the output, denoted as *X*, that is, choose to establish a connection with the network.

The possible formulas are as follows:

(8) $$\begin{array}{c} X=\min\left\{X\left({\bar{U}}_{RT}\right),X\left({\bar{U}}_{B}\right)\right\}. \end{array}$$

Since each input variable has three fuzzy levels, there are three 3 × 3 = 9 fuzzy rules, and all fuzzy rules are listed in Table 1.

Since the output of the input variable after fuzzy inference is still a fuzzy variable, it is necessary to obtain the exact output value of *i* by default. Here, the most commonly used priority method is used for defect classification. The formula is as follows:

(9) $$\begin{array}{c} \text{Fuzzy}\_\text{out}=\frac{\sum_{i}w_{i}M_{i}}{\sum_{i}M_{i}}. \end{array}$$

For nonimmediate services, the service program function U and the balance of service transmission time B are used as input variables in the fuzzy logic system, and the function of the average transmission time of nonimmediate services can be set as follows:

(10) $$\begin{array}{c} U_{\text{NRT}}\left(j\right)=\exp\left(\frac{T_{\text{NRT}\_\text{AVG}}-T_{\text{NRT}}\left(j\right)}{1+{\sqrt{T_{NR\_\text{AVG}}/T}}_{\text{NRT}\_\text{MAX}}}\right). \end{array}$$

Similar to real-time services, the network provisioning function of nonreal-time services is also expressed as follows:

(11) $$\begin{array}{c} U_{B}=\frac{\left[\sum_{i=1,i=j}^{K}\left(x_{i}/W_{T,I}\right)+\left(x_{j}+W_{j,m}/W_{T,j}\right)\right]}{k\left[\sum_{i=1,i=j}^{k}{\left(x_{i}/W_{T,i}\right)}^{2}+\left(x_{j}+W_{j,m}/W_{T,j}\right)\right]}. \end{array}$$

In the fuzzy logic system, the function *U* and the separation function B in the transfer time are used as input variables, and then, fuzzy logic processing is performed to realize the function of the input variables. First, the input variables are normalized. For the use of function *U*, the better the normalization formula, the smaller the use function.

(12) $$\begin{array}{c} {\bar{U}}_{NRT}=\frac{U_{\max}-U_{j}}{U_{\max}-U_{\min}}. \end{array}$$

After the input variables are fused, the initial value of the fusion is determined according to the fuzzy inference criterion.

(13) $$\begin{array}{c} X=\min\left\{X\left({\bar{U}}_{\text{NBT}}\right),X\left({\bar{U}}_{B}\right)\right\}. \end{array}$$

Since each input variable has three fuzzy levels, there are three 3 × 3 = 9 inference criteria, and all fuzzy rules are listed in Table 2.

Because the output basis of the output variable after fuzzy inference is the fuzzy variable, it is necessary to obtain the exact output amount by default. Here, the focus method is used for deficit classification. The formula is as follows:

(14) $$\begin{array}{c} \text{Fuzzy}\_\text{out}=\frac{\sum_{i}w_{i}M_{i}}{\sum_{i}M_{i}}. \end{array}$$

Considering the original vertical network transmission process, the problem lies in the improper setting of the network scanning circuit: on the one hand, if the network scanning cycle is too small, although a suitable wireless network can be found in time, the network identification time will be shortened. The power consumption of the terminal increases. If the network scanning time is too long, the terminal will not be able to detect a suitable wireless network in time, which increases the delay of network switching and affects the user's network experience. The terminal energy consumption dynamically adjusts the network scanning period, that is, if the signal strength in the current network is low, it means that the current status of the access network is poor, and the terminal has a greater demand for network replacement. Therefore, it is necessary to speed up the network scanning speed and reduce the corresponding scanning time. The calculation method is as follows:

(15) $$\begin{array}{c} T_{\text{CAV}}=T_{\max}-\left(T_{\max}-T_{\min}\right)\left(\left(1-\frac{{\text{RSS}}_{\text{can}}}{{\text{RSS}}_{tk}}\right)+\frac{V_{\text{can}}}{V_{\max}}\right). \end{array}$$

The parameters are blurred. The vomiting of input parameters is called parameter fuzzification. After processing the parameters at the connection point, the connection degree of the fuzzy amount is obtained, and it has the following characteristics:

(16) $$\begin{array}{c} u_{F}:U⟶\left[0,1\right]. \end{array}$$

When a decision-making problem arises in objective reality without giving exact information, decision-makers often use vague information and statistical research results or existing experience as references to set unclear rules and then obtain unclear results. The unwritten rule uses “If-So,” and the simple parameters are as follows:

(17) $$\begin{array}{c} \text{if}\;X\text{ is }B,\text{ then }T\text{ is }C. \end{array}$$

The focus method is as follows: focus on the area is represented by the tangent axis and the fuzzy curve, which can be regarded as the center of the geometric figure. Note that *u* is the precise value obtained by the final “translation” as follows:

(18) $$\begin{array}{c} u_{0}=\frac{\int uu_{F}\left(u\right)au}{\int u_{F}\left(u\right)du},\;u\in U. \end{array}$$

In objective reality, if the research object is scattered, the calculation formula is as follows:

(19) $$\begin{array}{c} u_{0}=\frac{\sum_{i=j}^{N}u_{j}u_{f}\left(u_{j}\right)}{\sum_{j=1}^{N}u_{F}\left(u_{j}\right)},\;u\in U. \end{array}$$

The weighted average coefficient method is as follows: the weighted average formula is mainly used to obtain the following:

(20) $$\begin{array}{c} U_{0}=\frac{\sum_{i=1}^{N}k_{j},u_{j}}{\sum_{j-1}^{N}u_{j}}. \end{array}$$

The maximum connection method is as follows: We select the element with the highest number of connections in the connection as the precise output. The calculation formula is as follows:

(21) $$\begin{array}{c} u_{F}\left(u_{0}\right)\geq u_{F}\left(u\right),\;u\in U. \end{array}$$

The application of formula (21) requires that the attribute function curve is a uniform curve. If the attribute function curve is a flat step curve, the element with the highest correlation is not clear, and all the corresponding elements are summarized and communicated. The calculation formula is as follows:

(22) $$\begin{array}{c} u_{0}=\frac{1}{N}\sum_{j=1}^{N}u_{j},u_{j}\in U. \end{array}$$

If *x* is the input signal, *w* is the weighted value corresponding to *x*; *n* is the number of input signals; and it is the value obtained by multiplying and accumulating the input signal vector and the weight vector. This is the final result after receiving the activation signal. The function *f* is 0, which is the following calculation formula:

(23) $$\begin{array}{c} O=f\left(\sum_{i=1}^{N}x_{i}\cdot w_{i}\right). \end{array}$$

If the sigmoid function is used as the activation function, the calculation formula is as follows:

(24) $$\begin{array}{c} f\left(x\right)=\frac{1}{1+e^{-x}}. \end{array}$$

If the step function is used as the activation function, the calculation formula is as follows:

(25) $$\begin{array}{c} f\left(x\right)=\left\{\begin{array}{cc} 0, & \text{if }x>0,\\ 1, & i\text{f }x\geq 0. \end{array}\right. \end{array}$$

The linear activation function is the simplest activation function, and its calculation formula is as follows:

(26) $$\begin{array}{c} f\left(x\right)=x. \end{array}$$

### 3.3. Supply Chain Model Design

**Figure 3:**
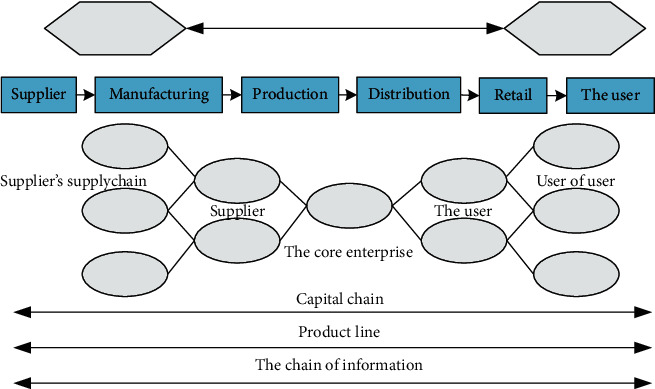
Supply chain network structure model.

The supply chain model must analyze the objective activities of the supply chain, introduce these activities and the entire supply chain system from one or more perspectives, and analyze the various activities introduced in the supply chain model to evaluate the results. The supply chain model takes many factors into consideration. The company in the actual supply chain contains a lot of content, such as the company's supply chain suppliers, manufacturers, large-scale integrated sellers, and retailers. Many members of the company must consider these to the end consumer. The supply chain members are integrated into the supply chain model, and the requirements of objective supply chain operation are accurately introduced; logistics information and capital in the entire supply chain reflect the most important information in the supply chain. This has also become a problem to be taken care of in the supply chain model. The original supply chain structure model only introduced that the structure of the supply chain system is stable, but the supply chain system is not an economic system, and it is only qualitative and quantitative, because its economic quantity model can no longer meet the requirements of development. The best way to achieve this problem is to create an economic cybernetic model 1 and a system simulation model of the supply chain, as shown in Figure 3.

**Figure 4:**
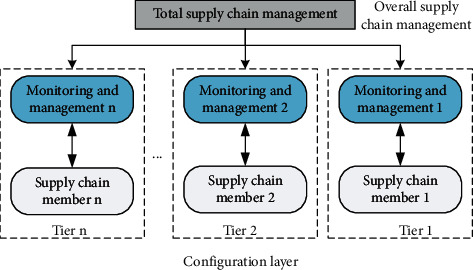
Overall framework of supply chain model.

The supply chain model should be as close as possible to the actual function of the supply chain. After describing the actual supply chain business in detail, it is assumed that the supply chain process reference model is accurate in describing supply. In order to reduce the complexity and difficulty of establishing a supply chain model, the supply chain system is divided into two layers: the first layer is the overall management of the supply chain system. The supply chain and the central company perform all these tasks at this level; the supply chain completes the overall goals and tasks of the supply chain. The second layer is the setting layer, which includes all companies in the supply chain and the corresponding control processing. The supply chain company executes the tasks assigned to the entire supply chain control processing department, and the relevant managers must control and manage the companies in the supply chain to ensure that the companies in the supply chain complete their tasks in accordance with the rules in the list. The details are shown in Figure 4.

**Figure 5:**
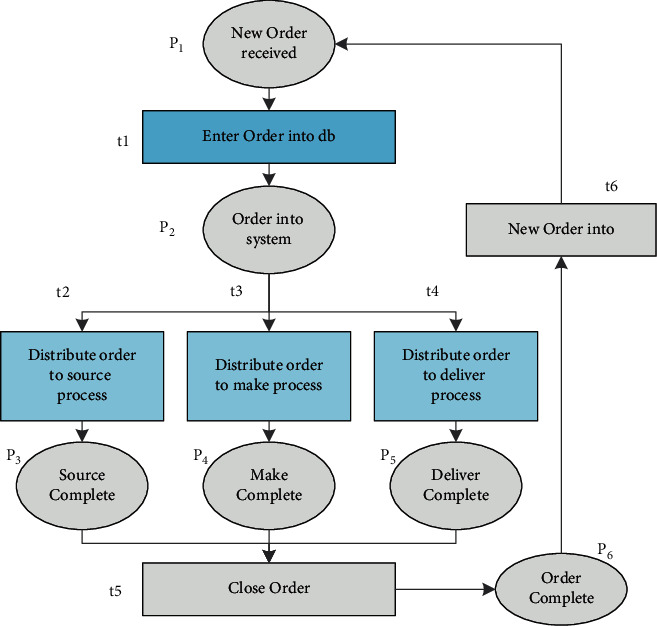
Petri net model of supply chain.

In this framework, each supply chain member in the setup layer and their respective control management constitute a relatively independent level. Therefore, the entire supply chain is composed of a one-to-one level in the entire supply chain control process. A hierarchical modeling method is used to reduce the complexity of the main model, and the complicated supply chain model is decomposed into several subchannel models, so as to avoid the explosion of the state area related to the performance analysis of the Petri network model, thereby analyzing difficult problems. The Petri network model of the supply chain is shown in Figure 5.

In the model, the ellipsis is used to indicate the location, and the field is used to indicate the change. Since the supply chain members apply the same process at each level of the setting layer, there are three main procurement, manufacturing, and distribution processes, each in the supply chain. The inventory and changes in the members are the same. We describe the importance of inventory, and the changes in the Petri grid model in the supply chain are listed in Table 3.

## 4. Practical Application of Supply Chain Efficiency Optimization

### 4.1. Supply Chain Workflow Setting

**Figure 6:**
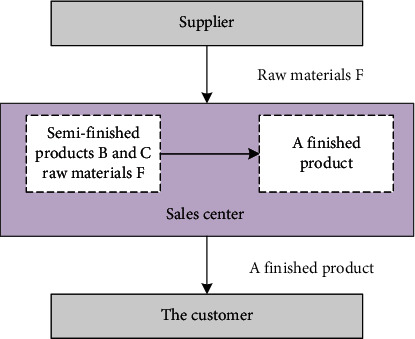
Optimized supply chain process.

In the previous case based on Petri networks' supply chain model and simulation, the assembly time of product A is a large part of the operation time of the supply chain because the sales center has no inventory, which represents the bottleneck problem of product A. The efficiency of the supply chain and the number of products are displayed in the sales center; after accepting the user's order, the finished product A is assembled; and the collection is delivered to the customer. The supply chain is shown in Figure 6.

Table 4 shows the specific conditions of the 7 database nodes and 5 transition nodes.

### 4.2. Analysis of Supply Chain Efficiency Optimization

Table 5 is the specific situation of the optimized GSPN model.

The analysis shows that the time unit of the number of symbols of each subsystem input subsystem corresponds to the number of transaction items leaving the warehouse at each time. At the same time, this subsystem contains all modifications. Therefore, the average execution time of the subsystem is the average of the supply chain. The execution time is shown in Table 6.

The local symbol in the supply chain model can describe whether the process of the supply chain is working as expected. If the token is in place, it means that the work process described by the location of the supply chain is operating, and the time required by the workflow in the supply chain system and the total running time in the supply chain need to be recorded. so each workflow records the relationship between the total running time of the supply chain, and the efficiency of the workflow is the overall conclusion drawn through calculations.

### 4.3. Supply Chain Efficiency Optimization Strategy

#### 4.3.1. Procurement Management Optimization

The first is to strengthen supplier management. With the development of the world economy, supply chains have become more complex and uncontrollable, making the supply chain vulnerable to economic crises, various disasters, social instability, and other factors. Suppliers cannot deliver products on time, so in order to reduce the risk of supply chain disruption, it is necessary to strengthen supplier management.

The second is to strictly control the delivery of goods. In order to avoid the problem of product homogeneity, companies should start with the source of the products. Platform sellers can attract brands and import products for a long period. One is that the company's purchasing department directly discusses cooperation with the brand owner and allows the brand owner to settle down. The second is the intensive development of long-term products; according to long-term theory, the integration of several small markets can generate energy to compete with the mainstream. High-quality sales or other platforms often follow the development of imports, so that many domestic consumers do not understand certain products. The platform can penetrate into the international market, and several products have been developed for a long time through social media.

#### 4.3.2. Logistics Management Optimization

One is an innovative logistic distribution model, and there are two innovative logistic models: one is logistic outsourcing; the original supply chain logistic model includes the following: overseas direct-advertising model, selling goods to consumers using postal parcels, express delivery, or logistics; this method is usually very expensive: with the help of customs and free trade zones, the bottom-up model has certain requirements for inventory estimation: build models in foreign countries, but the cost of building foreign warehouses in the early stage is very high; and the time is very long. The second is to establish a transnational e-commerce, third-party logistics, and third-party logistic cooperation alliance, established on the basis of equalization of interests with other companies, in order to reconcile its own logistic deficiencies and adopt a joint and win-win situation.

The second is to improve the logistic guarantee mechanism. There are two ways to improve the logistic guarantee mechanism: the first is to invest in the construction of a reverse logistic tracking system, which should be able to integrate. With the help of this system, supply chain import companies can understand the user ID, return status, and cost of reverse logistics. Users can also evaluate the cost and time required for returns.

#### 4.3.3. After-Sale Service Optimization

The first is to draft specific laws and regulations to protect the rights and interests of consumers, and choose the source solutions for consumer complaints. We strengthen the formulation and implementation of relevant laws and regulations, so that consumers can have legal support and protection when they consume or complain, so that consumers can purchase online with greater peace of mind and peace of mind. The second is to strengthen international exchanges and cooperation and establish an international protection mechanism for cross-border online consumers. We effectively protect the vital interests of consumers.

#### 4.3.4. The Overall Level of the Supply Chain Is Improved

One is to improve the level of information in the supply chain. Artificial intelligence can enhance user experience, and advanced artificial intelligence technology can improve user experience, and increase efficiency and model improvements.

The second is to train professionals in the supply chain. Talents are the biggest competitiveness for all companies to continue to develop. Compared with domestic e-commerce, the import supply chain contains more complex components, which makes the necessary professional knowledge more extensive. The theoretical content of students is integrated into social practice, and students must be able to participate more in the practice of supply chain companies.

## 5. Conclusions

In short, the cognitive radio system proposed in this study can receive network-related messages by observing the surrounding wireless network environment, and can receive internal communication tasks and emergency communication tasks based on historical data and prediction of future relationships. Through the access control process, we make an appropriate strategy and finally complete the communication function of the system.

## Figures and Tables

**Table 1:** Fuzzy rules in real-time business.

Rule number	Input		Output
	*X*(*Ukt*)	*X*(*Ub*)	*X*
1	*M*	*M*	*M*
2	*M*	*H*	*M*
3	*M*	*L*	*M*
4	*H*	*M*	*M*
5	*H*	*H*	*H*
6	*H*	*L*	*H*
7	*L*	*M*	*M*
8	*L*	*H*	*H*
9	*L*	*L*	*L*

**Table 2:** Fuzzy rules for nonreal-time business.

Rule number	Input		Output
	*X*(*Unrj*)	*X*(*Ur*)	*X*
1	*M*	*M*	*M*
2	*M*	*H*	*M*
3	*M*	*L*	*M*
4	*H*	*M*	*M*
5	*H*	*H*	*X*
6	*H*	*L*	*L*
7	*L*	*M*	*H*
8	*L*	*H*	*M*
9	*L*	*L*	*H*

**Table 3:** The place and meaning of the CTPN model of supply chain.

Treasury		Meaning	Change		Meaning
*P*1	NewOrderReceved	Receive new order	*T*1	Enter order nto db	Open the order library
*P*2	Order in systm	Open the order system	*T*2	Distribute ordeto source process	Assign order to purchasing program
*P*3	Source compete	End of purchase	*T*3	Distribute ordeto make process	Assign order to production program
*P*4	Make compete	End of production	*T*4	Distribute ordeto delver process	Assign items to delivery procedures
*P*5	Deliver compete	End of delivery	*T*5	Cose order	End of order
*P*6	Order compete	End of order	*T*6	New order	Receive new order

**Table 4:** Places and changes in the optimized model.

Treasury	Meaning	Change	Meaning	Delay factor
*P*1	Receive customer order	*T*1	Finished product A is produced	7
*P*2	Raw material supplier receives order	*T*2	Integrated delivery of raw material suppliers	3
*P*3	Raw materials arrive at the sales center	*T*3	Packaging of finished product A	4
*P*4	Semifinished product inventory	*T*4	Delivery of finished product A	3
*P*5	Semifinished product inventory	*T*5	Customer sends order information	5
*P*6	Finished product A packaging end			
*P*7	Customer receives finished product A			

**Table 5:** The reachable flag set of the optimized GSPN model.

Logo	*P*1	*P*2	*P*3	*P*4	*P*5	*P*6	*P*7
*M*1	0	0	0	0	0	0	0
*M*2	0	0	0	1	1	1	1
*M*3	1	0	1	0	0	0	0
*M*4	0	1	0	1	1	1	1
*M*5	0	0	1	0	0	0	0

**Table 6:** The average number of tokens in the optimized subsystem library.

*P*1	*P*2	*P*3	*P*4	*P*5	*P*6	*P*7
*P*(*M*(*P*1)) − 1	0.287	0.18	0.477	0.477	0.287	0.144

## Data Availability

The data used to support the findings of this study are available from the corresponding author upon request.
